# Two Cases of Children With Black Hairy Tongue and Tooth Discoloration Caused by Antibacterial Agents

**DOI:** 10.7759/cureus.58354

**Published:** 2024-04-15

**Authors:** Mayumi Yamada, Kensuke Shoji, Tetsuya Fukuda, Chiaki Tao, Shota Myojin, Hideki Ogiwara, Kenichi Usami, Jumpei Saito

**Affiliations:** 1 Department of Pharmacy, National Center for Child Health and Development, Tokyo, JPN; 2 Division of Infectious Diseases, National Center for Child Health and Development, Tokyo, JPN; 3 Department of Neurological Surgery, National Center for Child Health and Development, Tokyo, JPN

**Keywords:** meropenem, vancomycin, linezolid, tooth discoloration, black hairy tongue

## Abstract

Black hairy tongue (BHT) is a lesion in which the filiform papillae of the tongue are significantly extended by hyperkeratosis, thereby giving the tongue a hairy appearance. Here, we report two rare cases of children with BHT and tooth discoloration caused by antimicrobial agents. Case 1: A four-year-old female patient received intravenous linezolid after spinal surgery, and BHT developed on day eight of treatment. Subsequently, the patient developed teeth discoloration. Linezolid was continually administered for 50 days, and BHT and teeth discoloration improved 10 days after the end of linezolid treatment. Case 2: A two-year-old male patient with a brain abscess received intravenous meropenem and vancomycin. On the fourth day of treatment, BHT developed, and teeth discoloration was subsequently observed. Antibiotic therapy was continued for 82 days, and BHT and tooth discoloration improved 20 days after the treatment was discontinued.

## Introduction

Black hairy tongue (BHT) is a lesion in which the filiform papillae of the tongue are significantly extended by hyperkeratosis, thereby giving the tongue a hairy appearance [1]. BHT is caused by various factors, including poor oral hygiene, medications (e.g., antibiotics, oral iron supplements, steroids, anticancer drugs, and antipsychotics), luxury grocery items (e.g., smoking, alcohol, coffee, and tea), immunocompromised conditions caused by different diseases (e.g., human immunodeficiency virus infection and cancer), and radiation therapy to the head and neck region [2]. Tooth discoloration refers to the change in tooth color, and it can be caused by various factors including antibiotics [3]. BHT is a benign condition that generally resolves spontaneously. However, the reversibility of antibiotic-induced tooth discoloration depends on the type of antibiotics [3]. Only a few case reports of concomitant BHT and tooth discoloration are recorded, all in pediatric cases. Furthermore, information on the clinical course and prognosis of these conditions is limited.

Here, we report two cases of children with BHT and tooth discoloration that improved after antibiotic discontinuation.

## Case presentation

Case 1

**Figure 1:**
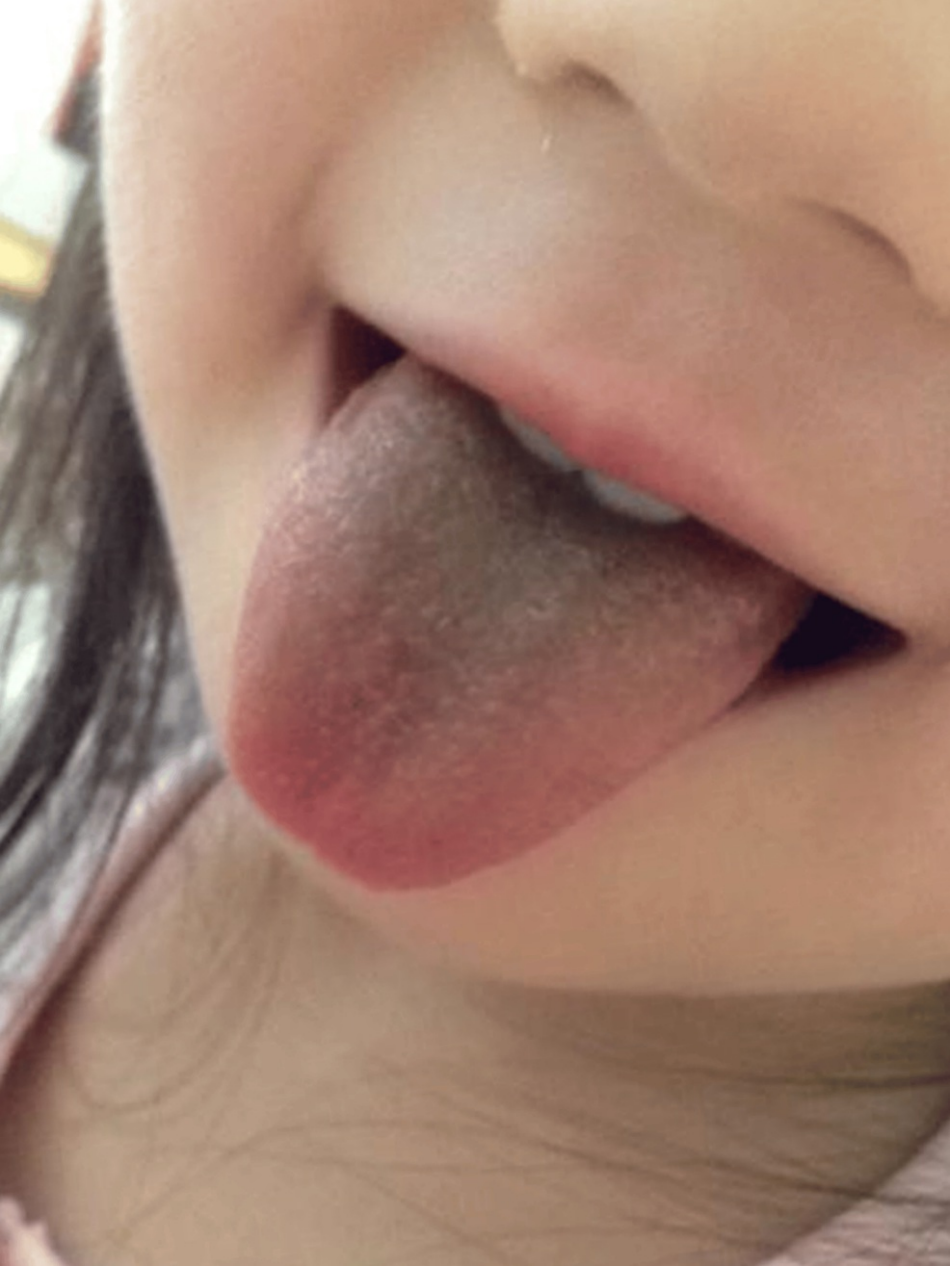
Black hairy tongue during linezolid therapy in case 1. Day eight of linezolid therapy.

**Figure 2:**
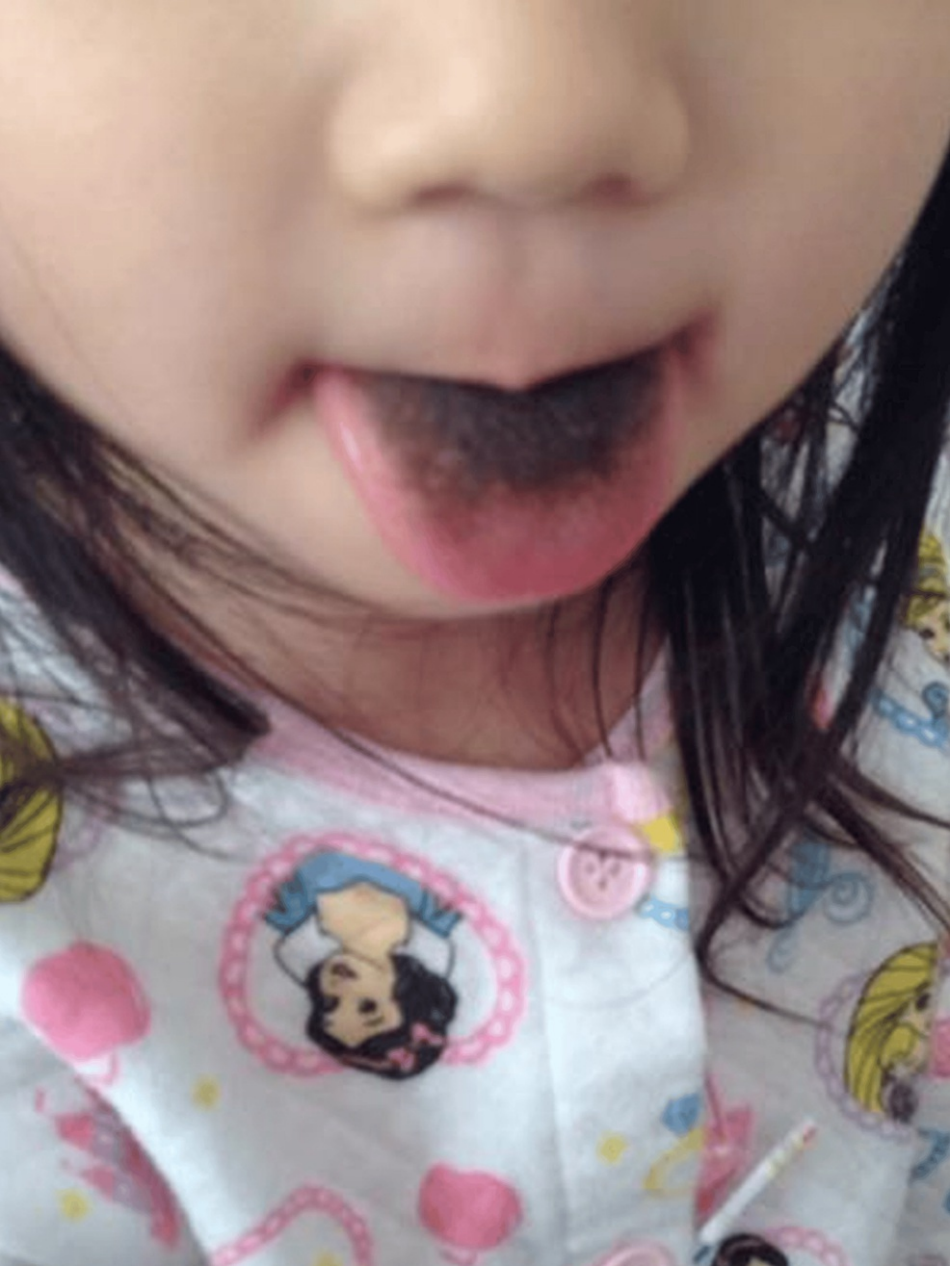
Black hairy tongue during linezolid therapy in case 1. Day 35 of linezolid therapy.

**Figure 3:**
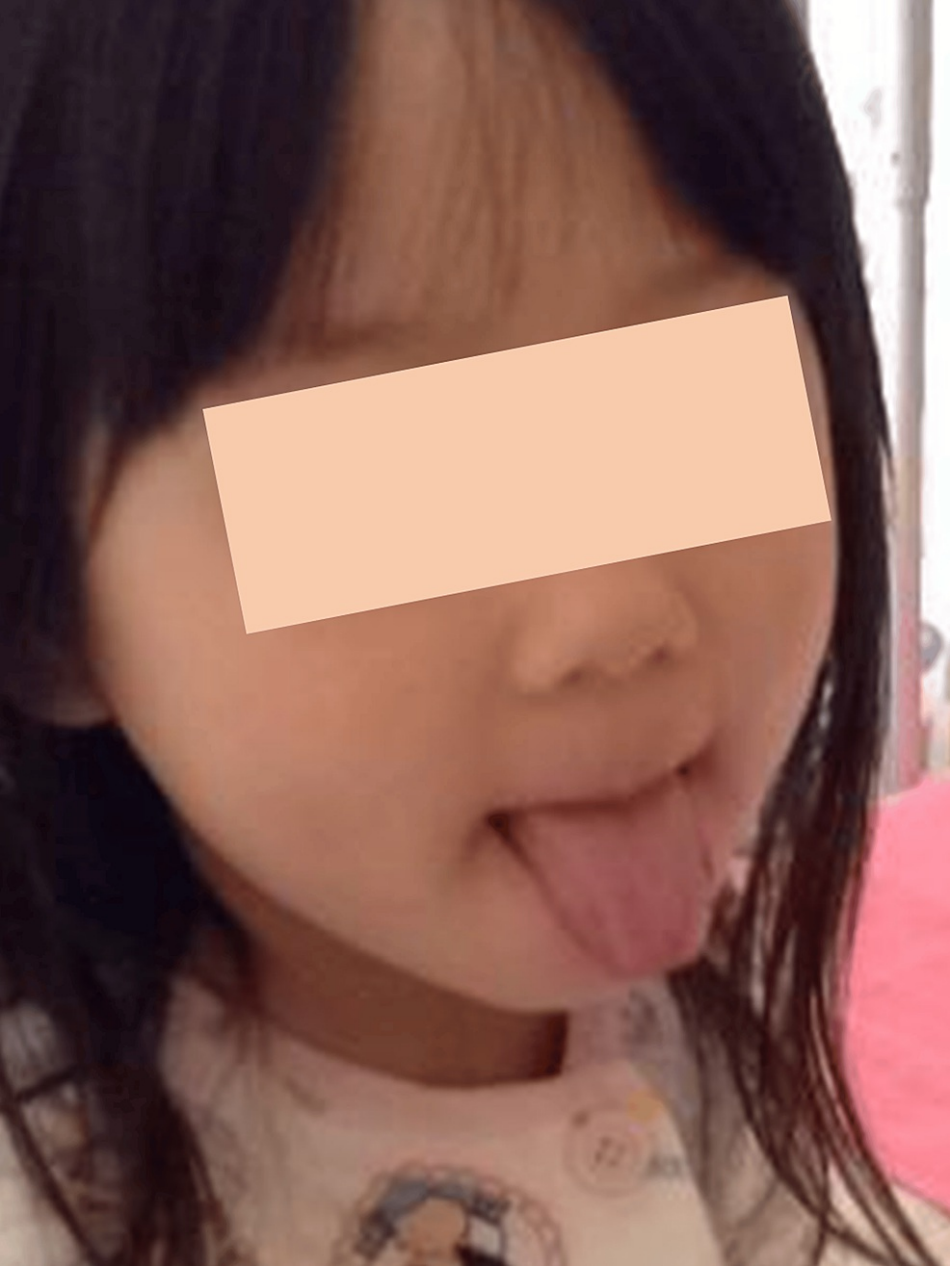
Black hairy tongue during linezolid therapy in case 1. Day 11 after treatment completion.

A four-year-old female patient with a neurogenic bladder was admitted to the hospital due to surgery for a thoracolumbar intramedullary tumor and syringomyelia. A tumor biopsy and cutaneous sinusectomy were performed. Cefotaxime, which was administered as a prophylaxis against surgical site infection, was continually administered after surgery as central nervous system infection was suspected. On day 19 of treatment, the patient developed cyanosis and tachycardia, with decreased oxygen saturation levels. Cefotaxime allergy was suspected. Thus, cefotaxime was switched to vancomycin (VCM). On day 31 of VCM treatment, VCM was switched to intravenous linezolid (LZD) because of neutropenia possibly caused by VCM. On day eight of LZD, the center of the patient’s tongue turned partially blue or light gray (Figure 1). LZD-induced BHT was suspected because no concomitant medications could have caused BHT. The plan was to continue the antimicrobial therapy until the tumor lesion could be evaluated over time using MRI to determine whether it was growing or not. The patient did not present with oral pain or taste disorder, and her food intake did not change. LZD was continually administered due to limited antibiotic availability. Subsequently, the partial blue or light gray lesions on the tongue gradually turned black (Figure 2). Tooth discoloration was observed near the gums and was reversible with tooth brushing. However, some mild staining on the lower teeth remained. LZD was used for 50 days. Tongue coloration and tooth discoloration improved approximately 10 days after the end of LZD (Figure 3). BHT did not improve with tongue brushing until LZD was discontinued. The patient was discharged on the last day of the antibiotic therapy; no recurrence of BHT or tooth discoloration was observed 48 days posttreatment.

Case 2

**Figure 4:**
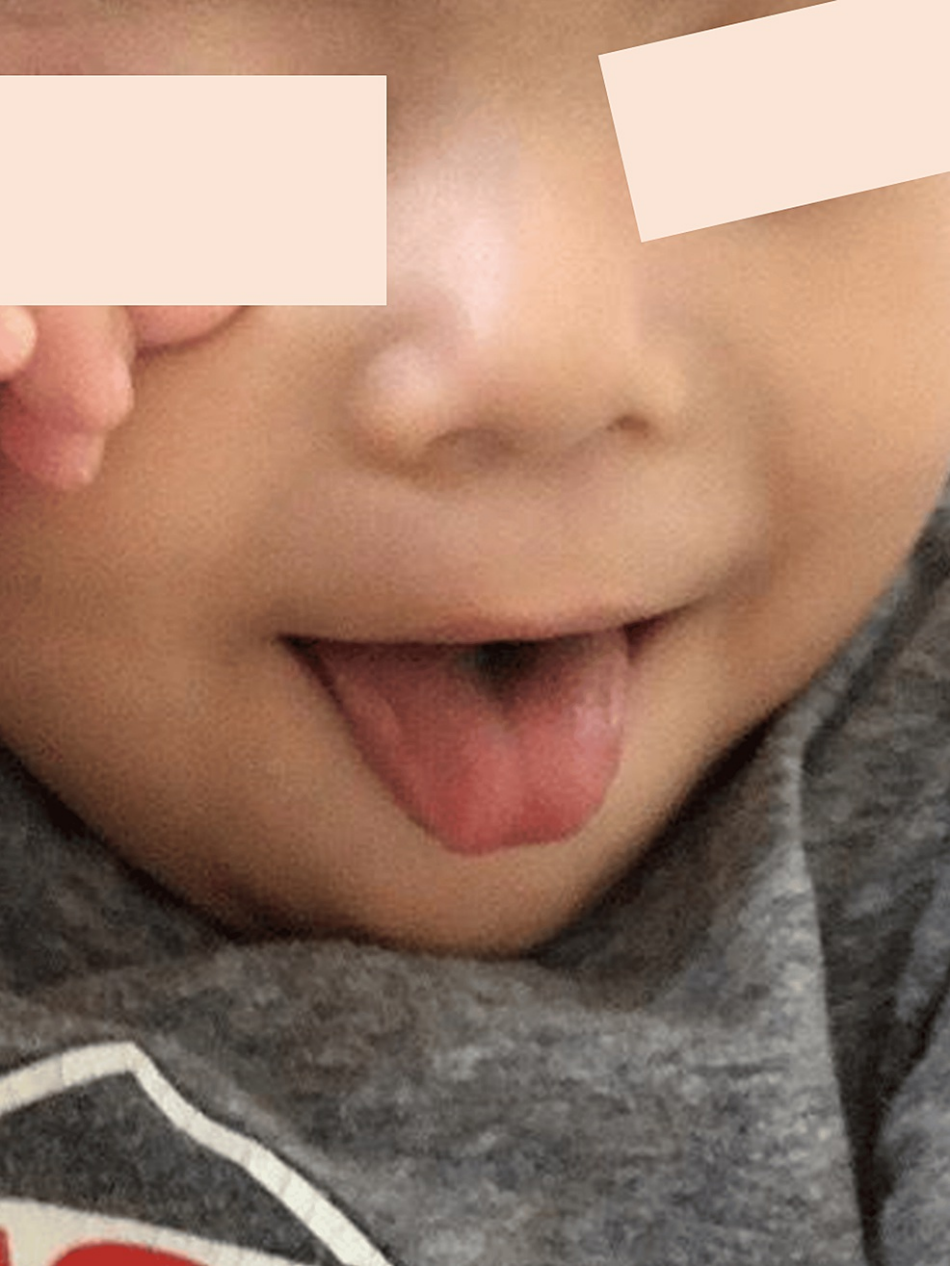
Black hairy tongue during meropenem and vancomycin therapy in case 2. Day four of meropenem and vancomycin therapy.

**Figure 5:**
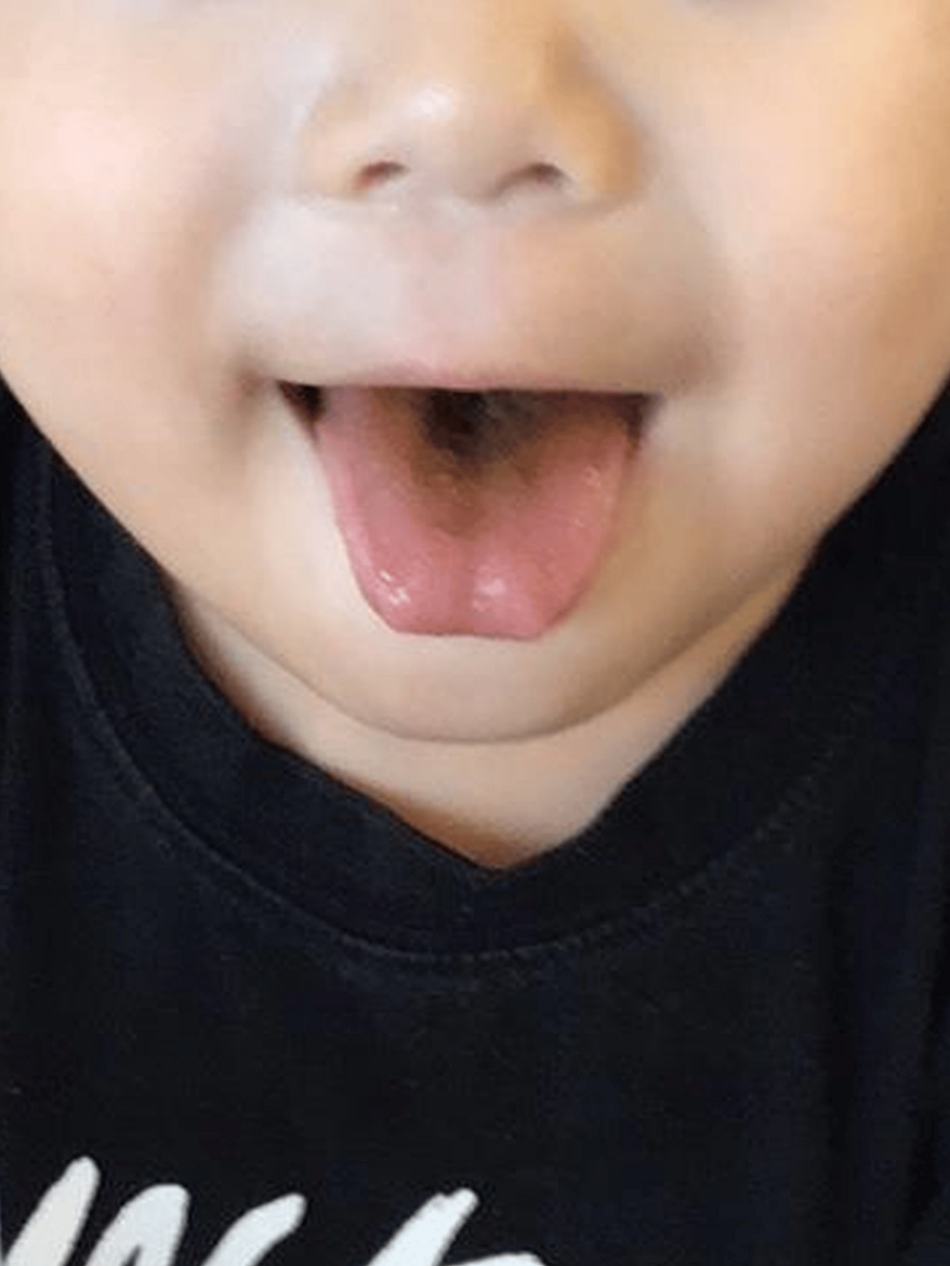
Black hairy tongue during meropenem and vancomycin therapy in case 2. Day 16 of meropenem and vancomycin therapy.

**Figure 6:**
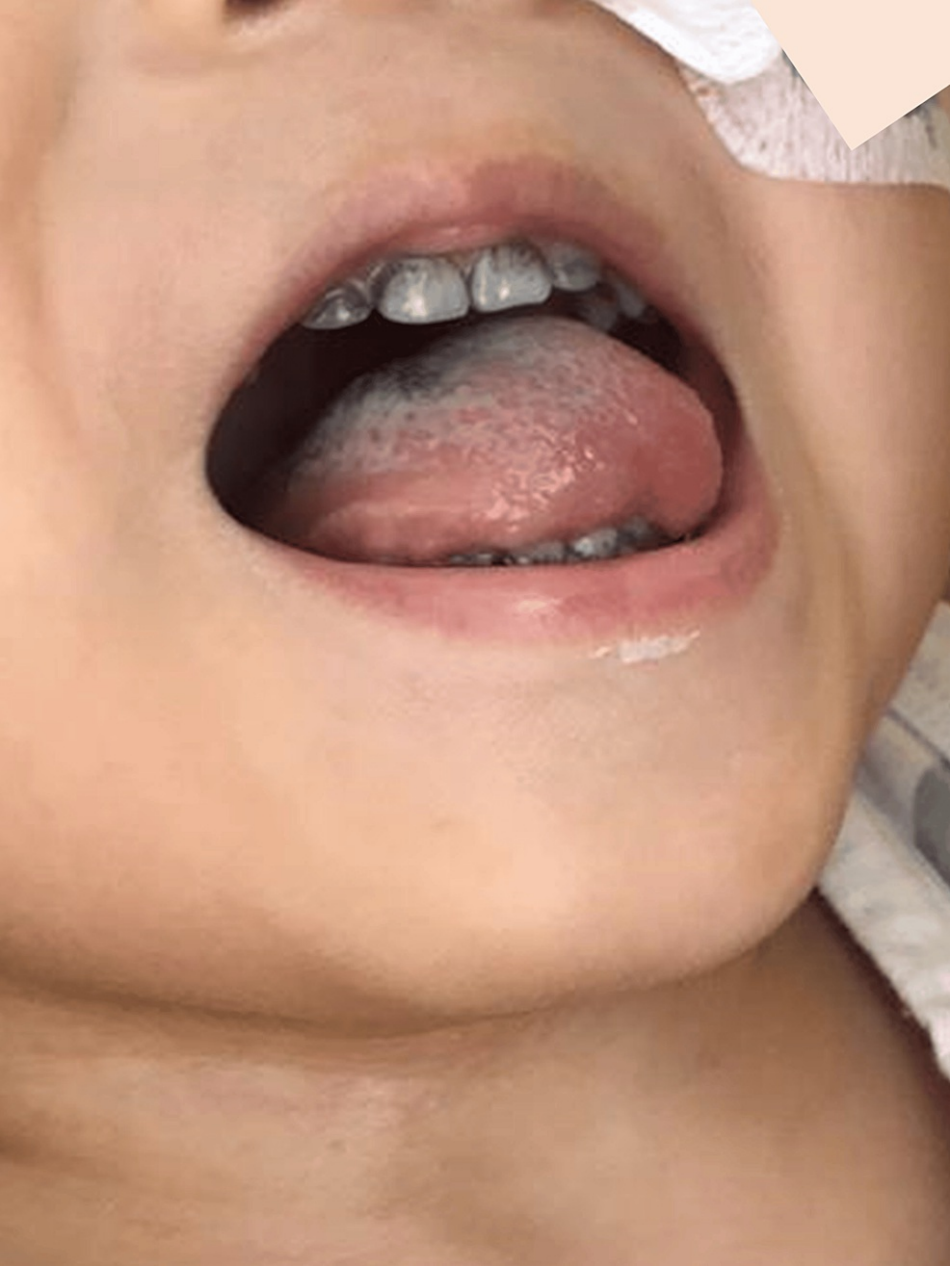
Tooth discoloration during meropenem and vancomycin therapy in case 2. Day six after treatment completion.

**Figure 7:**
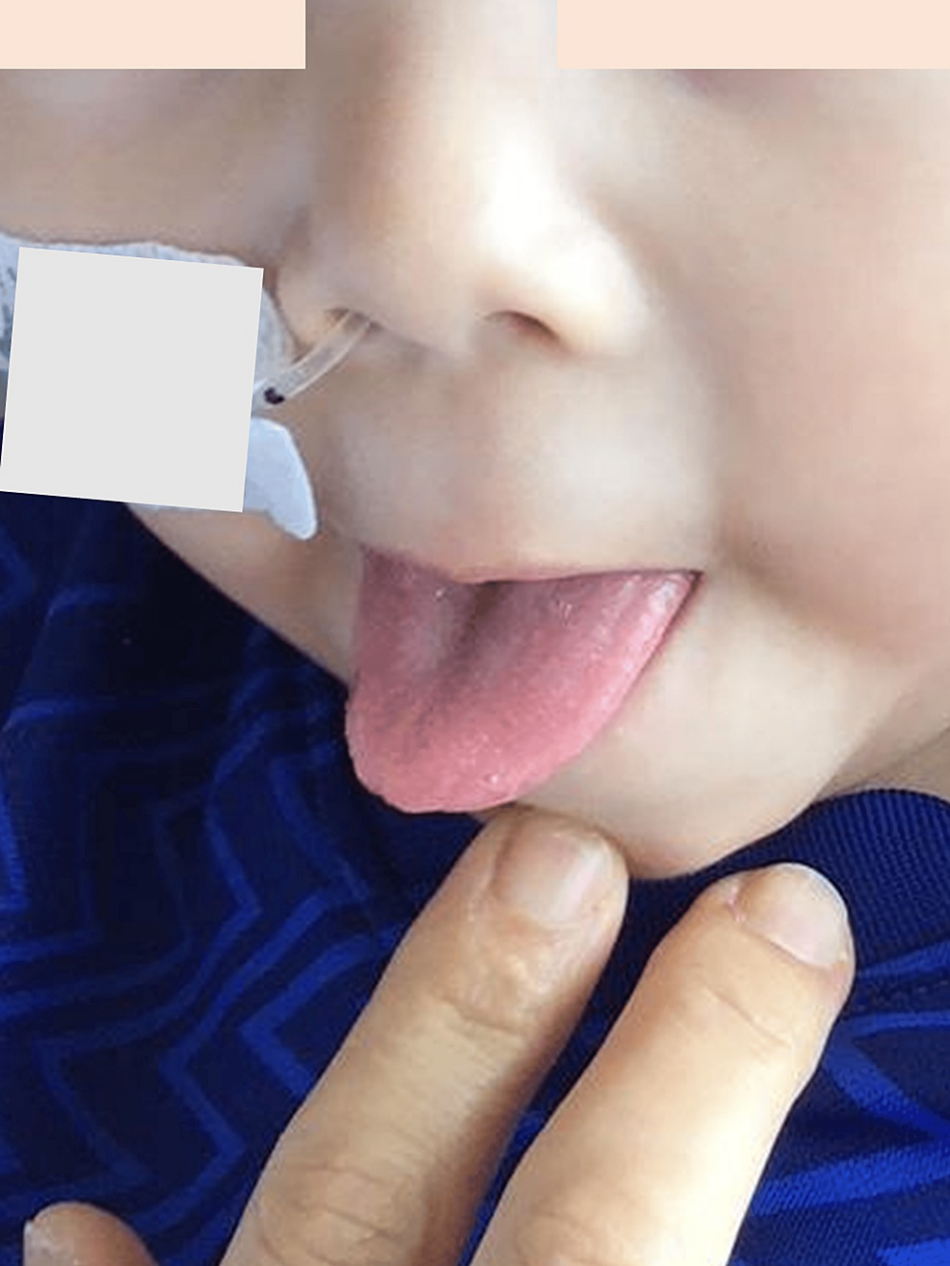
Black hairy tongue during meropenem and vancomycin therapy in case 2. Day 26 after treatment completion.

**Figure 8:**
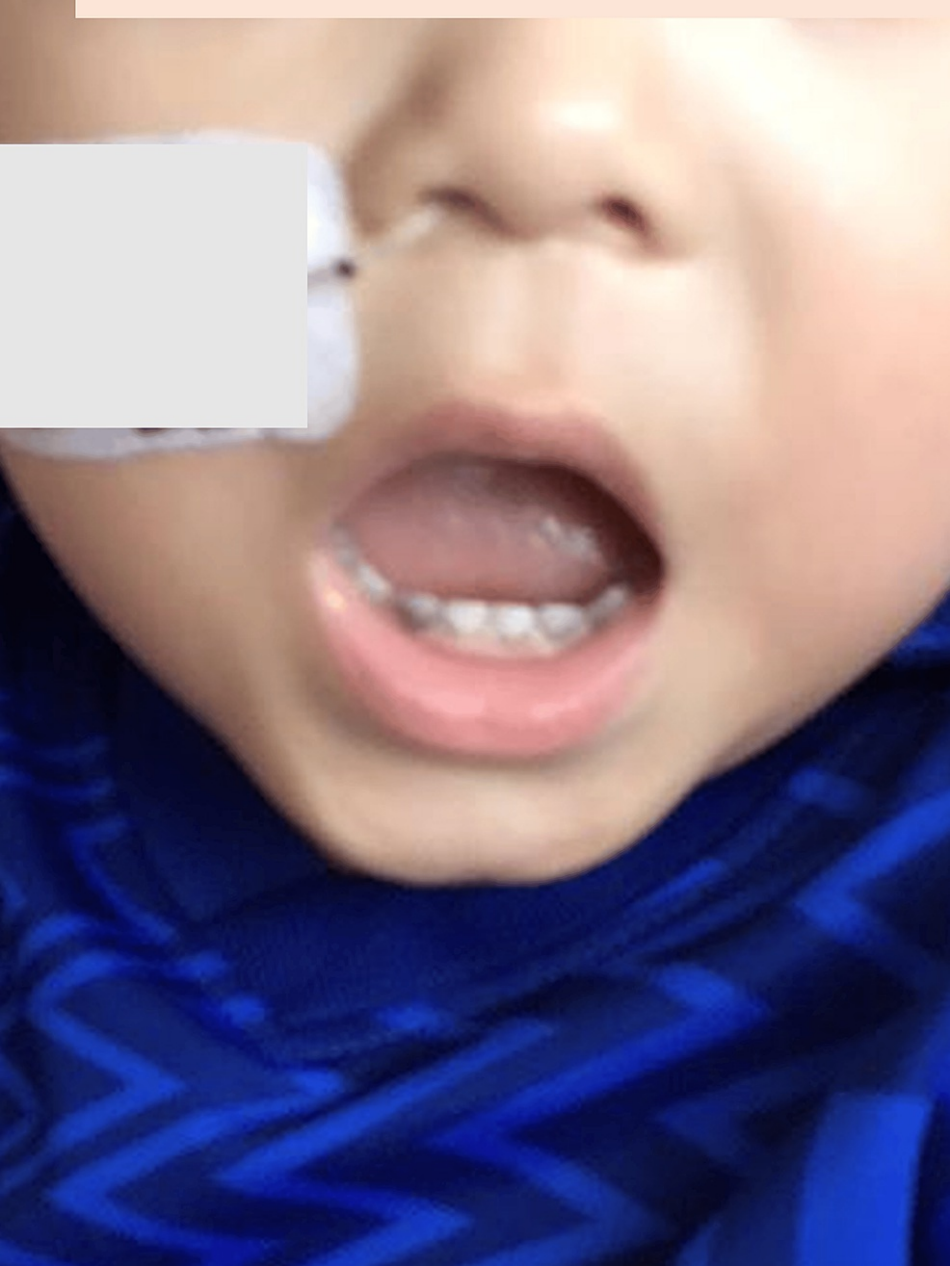
Tooth discoloration during meropenem and vancomycin therapy in case 2. Day 26 after treatment completion.

A two-year-old male patient with congenital esophageal stricture was admitted to the hospital due to a febrile seizure. MRI revealed a brain abscess in the right parietal lobe. Therefore, treatment with cefepime, VCM, and metronidazole was initiated. Abscess drainage was performed on day two of antibiotic treatment, and *Streptococcus intermedius* was detected from the abscess. Based on the sensitivity results, cefepime and VCM were discontinued, and treatment with cefotaxime was initiated. On day 18 of cefotaxime and metronidazole therapy, the patient developed a fever and vomiting. Drug allergy was suspected, and the antibiotics were changed to intravenous meropenem (MEPM) and VCM. Symptoms of fever and vomiting improved quickly post-drug change. On day four of MEPM and VCM therapy, the central part of the tongue partially turned blue or light gray (Figure 4). Because there were no complaints of oral pain or taste disturbance, MEPM and VCM were continually administered. Subsequently, the partial blue or light gray lesions on the tongue gradually turned black (Figure 5), and teeth staining also developed (Figure 6). MEPM and VCM treatment was successfully completed for 82 days until the brain abscess resolved in the MRI. Tongue coloration improved approximately 20 days after the end of the treatment (Figure 7). Tooth discoloration also improved with tooth brushing. However, slight staining remained (Figure 8). In addition to antibiotics, ferrous citrate can cause BHT and tooth discoloration. However, treatment with ferrous citrate was started on day 26 of MEPM and VCM therapy. By then, BHT and tooth discoloration had already been observed, Furthermore, BHT and tooth discoloration improved even though ferrous citrate was continually administered. The patient was discharged 15 days post-antibiotic therapy and no recurrence of BHT or tooth discoloration was observed 41 days posttreatment.

## Discussion

Here, we present two cases of BHT and tooth discoloration that might be attributed to the use of antimicrobial agents. Both patients had no symptoms except for color change, and the color improved spontaneously after the end of antibiotic therapy.

BHT was first described by Amatus Lusitanus in 1557 [1], and several cases of BHT have been reported to date [2]. Although the pathophysiology of BHT is not completely understood, it is believed to be caused by poor desquamation of the dorsal surface of the tongue [1]. The resulting enlargement and elongation of the filiform papillae give the appearance of hair growth on the tongue [1,2]. It is speculated that the coloration is caused by the accumulation of residues of bacteria, fungi, food, coffee, tea, tobacco, and other substances in the enlarged filiform papillae [1]. Further, it may be caused by porphyrin production by chromogenic bacteria or yeasts [4]. However, no specific bacteria or yeast organisms have been identified as possible causes of BHT, and the definitive cause remains unknown. Although BHT rarely requires aggressive treatment because they heal spontaneously with discontinuation of the suspected agents, bacterial culture could be useful in the future to determine the cause and as a pathogenic marker of infection.

Drug-induced BHT has been reported with the use of antibiotics, medications causing xerostomia (such as olanzapine and chlorpromazine), and anticancer drugs (such as erlotinib) [4]. These drugs can cause oral dysbiosis, such as dry mouth and immunosuppressed states. Some reports have shown that antibiotics including piperacillin/tazobactam [2], levofloxacin [2], moxifloxacin [1], cefazolin [4], penicillin [5], amoxicillin/clavulanic acid [6], metronidazole [6], erythromycin [7], minocycline [8], doxycycline [9], imipenem/cilastatin [10], ceftriaxone [11], MEPM [12], and LZD [2,12] can induce BHT. In the current report, the causes of BHT were LZD in case 1 and MEPM or VCM in case 2. To our knowledge, this is the first report on BHT caused by combined MEPM and VCM. Hence, it is important to be knowledgeable that different antimicrobial agents can cause BHT.

Drug-induced tooth discoloration has been reported with the use of antibiotics, chlorhexidine, oral iron supplements, and fluoride [3]. Antibiotic-induced tooth discoloration has been reported with the use of tetracycline [13], amoxicillin [14], imipenem [15], ciprofloxacin [16], and LZD [17]. The prognosis of antimicrobial tooth discoloration varies with each antibiotic. The mechanism of tooth discoloration caused by tetracycline is attributed to deposition in dentin by chelating calcium ions, and the discoloration is permanent [13]. However, the discoloration caused by amoxicillin is reversible [14]. In the current cases, tooth discoloration, which could have been caused by LZD, MEPM, or VCM, was reversible. The mechanism of tooth discoloration caused by LZD is not yet clear. Nevertheless, two potential mechanisms are suggested [17]. First, the oral microflora could have been altered, thereby causing the attachment of chromogenic microorganisms to the tooth surface. Second, the condition was attributed to the structural affinity of LZD for teeth. In the report of Zou et al., the condition could be associated with the first mechanism. The incidence of tooth discoloration in children is higher than that in adults, which may be caused by different oral bacterial flora [17]. However, the potential mechanism of tooth discoloration caused by MEPM or VCM has not been identified.

Further research should be performed to validate the pathophysiology of tooth discoloration caused by these antibiotics.

In the current cases, BHT and tooth discoloration improved after the end of antimicrobial therapy. The treatment for BHT and tooth discoloration discontinues the suspected agents, and oral care is potentially useful for prevention. If BHT or tooth discoloration develops, it is challenging to decide whether to discontinue the therapy. Similar to our cases, there are other cases in which BHT and/or antimicrobial tooth discoloration was reversible [2,17]. These reports and our cases may indicate that it is acceptable to continue antibiotic therapy if BHT and/or tooth discoloration develop if there are no other good alternative antibiotics. However, case reports of antimicrobial-induced BHT and tooth discoloration are limited, and more cases should be accumulated for further discussion. Both cases were followed up for approximately 40 days post-antibiotic therapy; however, they should be regularly checked for negative BHT and tooth discoloration.

## Conclusions

The two patients in this case report continually received antibiotic treatment even after the development of BHT and tooth discoloration. Moreover, their conditions improved after treatment completion. To our knowledge, this may be the first report on concomitant BHT and tooth discoloration caused by MEPM and VCM. In the current cases, interviews with the patients’ families helped in the early detection of adverse effects and longitudinal images. Hence, LZD, VCM, and MEPM therapy can be continued if antibiotic therapy is highly needed clinically and if issues such as oral pain and taste disturbance are not observed.

## References

[1] Kato M, Kobayashi T, Suzuki H (2022). Case of moxifloxacin-induced black hairy tongue. Am J Case Rep.

[2] Ren J, Zheng Y, Du H (2020). Antibiotic-induced black hairy tongue: two case reports and a review of the literature. J Int Med Res.

[3] Tredwin CJ, Scully C, Bagan-Sebastian JV (2005). Drug-induced disorders of teeth. J Dent Res.

[4] Kano Y (2023). Black tongue discoloration. JAMA.

[5] Nowak DA, Nowak BE (2022). Black hairy tongue: a spooky sign associated with penicillin therapy. Med J Aust.

[6] Sheikh Z, Khan AS, Khan S (2011). Lingua villosa nigra. Lancet.

[7] Pigatto PD, Spadari F, Meroni L, Guzzi G (2008). Black hairy tongue associated with long-term oral erythromycin use. J Eur Acad Dermatol Venereol.

[8] Hamad Y, Warren DK (2018). Black hairy tongue. N Engl J Med.

[9] Ramsakal A, Mangat L (2007). Images in clinical medicine. Lingua villosa nigra. N Engl J Med.

[10] Zhao S, Fan L, Feng J, Ma P (2020). Reversible black tongue: a little known side effect of imipenem/cilastatin and evidence for novel mode of action. J Clin Pharm Ther.

[11] Yi-Chun L (2018). Black tongue. Eur J Intern Med.

[12] Petropoulou T, Lagona E, Syriopoulou V, Michos A (2013). Teeth and tongue discoloration after linezolid treatment in children. Pediatr Infect Dis J.

[13] Sánchez AR, Rogers RS 3rd, Sheridan PJ (2004). Tetracycline and other tetracycline-derivative staining of the teeth and oral cavity. Int J Dermatol.

[14] Meyboom RH, Verduijn MM, Steenvoorden MG, Dekens-Konter JA, van Puijenbroek EP (1996). [Reversible tooth discoloration during oral use of antibiotics]. Ned Tijdschr Geneeskd.

[15] Scanlon N, Wilsher M, Kolbe J (1997). Imipenem induced dental staining. Aust N Z J Med.

[16] Lumbiganon P, Pengsaa K, Sookpranee T (1991). Ciprofloxacin in neonates and its possible adverse effect on the teeth. Pediatr Infect Dis J.

[17] Zou D, Xu P, Zhang Y, Lu C, Wang J, Leng B, Zhang W (2020). The first case of teeth discoloration induced by linezolid in children in China Mainland. J Infect Chemother.

